# Hepatitis C Virus Infection and Dialysis: 2012 Update

**DOI:** 10.5402/2013/159760

**Published:** 2012-12-17

**Authors:** Fabrizio Fabrizi

**Affiliations:** Division of Nephrology and Dialysis, Maggiore Hospital and IRCCS Foundation, Padiglione Croff, Via Commenda 15, 20122 Milan, Italy

## Abstract

Hepatitis C virus infection is still common among dialysis patients, but the natural history of HCV in this group is not completely understood. Recent evidence has been accumulated showing that anti-HCV positive serologic status is significantly associated with lower survival in dialysis population; an increased risk of liver and cardiovascular disease-related mortality compared with anti-HCV negative subjects has been found. According to a novel meta-analysis (fourteen studies including 145,608 unique patients), the adjusted RR for liver disease-related death and cardiovascular mortality was 3.82 (95% CI, 1.92; 7.61) and 1.26 (95% CI, 1.10; 1.45), respectively. It has been suggested that the decision to treat HCV in patients with chronic kidney disease be based on the potential benefits and risks of therapy, including life expectancy, candidacy for kidney transplant, and co-morbidities. According to recent guidelines, the antiviral treatment of choice in HCV-infected patients on dialysis is mono-therapy but fresh data suggest the use of modern antiviral approaches (i.e., pegylated interferon plus ribavirin). The summary estimate for sustained viral response and drop-out rate was 56% (95% CI, 28–84) and 25% (95% CI, 10–40) in a pooled analysis including 151 dialysis patients on combination antiviral therapy (conventional or pegylated interferon plus ribavirin).

## 1. Introduction

Hepatitis C virus (HCV) infection remains frequent in patient receiving long-term dialysis both in developed and less-developed countries. The natural history of HCV infection in dialysis patients remains incompletely understood; controversy continues even in patients with intact kidney function. Defining the natural history of HCV remains difficult for several reasons: the disease has a very long duration, it is mostly asymptomatic, and determining its onset may be difficult. Additional factors can modify the course including coinfection with HBV, HIV, and alcohol use. Because treatment is widely used, future natural history studies of chronic HCV may not be possible as easily documented onset of infection, that is, posttransfusion HCV, no longer occurs [1, 2].

Assessing the natural history of hepatitis C among patients on regular dialysis is even more problematic because of additional characteristics of this population. Nephrologists have been reluctant to perform liver biopsy due to concern about abnormalities in platelet function in uraemia. Amino-transferase activity is lower in patients with chronic renal failure than in nonuremic population, and this may hamper recognition of HCV-related liver disease. Although third-generation anti-HCV testing is specific and sensitive in patients with end-stage renal disease, earlier versions of anti-HCV testing have been less reliable in ESRD patients because of the blunted humoral immune response that occur with renal disease: a small proportion of ESRD patients have HCV viraemia in serum, but lack detectable anti-HCV [3].

Mortality is an identifiable complication of liver disease and a reliable end-point in the natural history of HCV-related liver disease. Recent evidence indicates that HCV plays a detrimental effect on survival in the dialysis population, but it remains unknown whether the elevated mortality risk because of HCV infection is only attributable to an increase in liver disease-related deaths.

## 2. Epidemiology

Hepatitis C virus is a blood-borne pathogen that appears to be endemic in many parts of the world. The World Health Organization (WHO) estimates that global prevalence of HCV infection averages 3% or around 170 million infected persons worldwide. However, population-based surveys are not available in many parts of the world, and prevalence estimates are based on testing of selected populations such as blood donors. Prevalence of confirmed serologic status for anti-HCV antibody in blood donors ranges from less than 0.1% in northern Europe to 0.5% in western Europe and North America. Higher rates have been reported from Brazil, Eastern Europe, the Mediterranean area, and parts of Africa and Asia (1%–5%) [4].

Soon after the discovery of HCV as the major cause of non-A, non-B hepatitis, HCV was recognized as the most important agent of liver disease among patients receiving long-term dialysis. As in the general population, the prevalence of HCV among dialysis patients varies worldwide, ranging from as low as 1% to as high as over 70%. It is important to emphasize that the prevalence of anti-HCV positive patients on long-term dialysis in northern Europe is below 5%, around 10% in most of southern Europe and the USA, between 10% and 70% in many countries of the developing world, including north Africa, Asia, and southern America. The risk of HCV transmission is almost only parenteral: before the 1990s, the main routes of transmission were blood product transfusion, intravenous drug use, or unsafe injection procedures. Since the systematic screening of blood products, the residual risk of transfusion HCV infection is extremely low. The most important routes of transmission of HCV in industrialized countries remain intravenous or nasal drug use, mother to child transmission, and unsafe medical or surgical procedures. The risk of sexual transmission is rather low. The most important risk factors for acquisition of HCV in dialysis patients include blood transfusions and total spent time on dialysis. Additional risks factors include intravenous drug use and a history of kidney transplantation. The dialysis-related risk is around 2%, varying according to the countries; healthcare-related transmission of HCV can be eradicated with the full adherence to infection control procedures designed to prevent transmission of blood-borne pathogens [4].

There is abundant information on prevalence and incidence rates of HCV infection among patients on long-term dialysis in developed countries, and several population-based surveys have been made to this aim [5–8]. On the contrary, evidence on epidemiology of HCV in dialysis patients from developing countries is poor and mostly based on single-center studies [9–15]. Since the introduction in the 1990s of the screening of blood donors, transmission of HCV by blood transfusions is now exceedingly rare in dialysis units in developed countries. A big decrease in the frequency of anti-HCV antibodies has been noted during the 1990s in several countries from western Europe as an example the frequency of anti-HCV antibody in Italy ranges now between 8% and 12% (around 30% in early 1990s) [16–19]. The prevalence of positive serologic status for anti-HCV antibody has apparently not changed significantly over the last years (8%–10%) among patients receiving long-term dialysis in the USA, according to the data collected from the Centers for Disease Control and Prevention (CDC) [20]. The evolution of the epidemiology of HCV among patients on long-term dialysis in other countries is still unclear. It is important to give emphasis to that the prevalence of HCV is highly variable from unit to unit within the same country, with recent reports from some dialysis units in the USA reporting prevalences above 20% [20].

## 3. HCV-Related Liver Disease in Dialysis Patients: Biochemical and Clinical Manifestations

HCV-related liver disease is mostly asymptomatic in patients on long-term dialysis. Some symptoms which typically occur in nondialysis patients with HCV (i.e., asthenia, cognitive impairment) are common in the dialysis population irrespective of their HCV serologic status. Biochemical evaluation of HCV infection in patients on long-term dialysis is inaccurate as serum aminotransferase values are typically lower in dialysis patients than the nonuremic populations. Dialysis patients who are HCV viraemic have aminotransferase levels greater than those without, although values are still within the “normal” range. In a series of 394 patients receiving regular haemodialysis in the greater Los Angeles area, serum aspartate and alanine aminotransferase levels were significantly higher in viraemic patients than in individuals with no detectable HCV RNA in serum: 23.8 (95% CI, 60.8–9.3) *versus* 17.1 (95% CI, 50.4–5.8) U/L (*P* = 0.009) and 14.4 (95% CI, 48.9–4.3) *versus* 9.8 (95% CI, 37.2–2.5) U/L (*P* = 0.008). Logistic regression analysis demonstrated a strong association between HCV viraemia and positive serologic status (*P* = 0.0001) and ALT activity (*P* = 0.01) [21].

Serum gamma glutamyl-transpeptidase (GGTP) were measured in a large cohort (*n* = 757) of patients receiving maintenance dialysis in northern Italy; serum GGTP were higher in HBsAg positive and/or anti-HCV positive patients than in HBsAg negative/anti-HCV negative patients on dialysis; 85.1 ± 184.1 *versus *25.8 ± 23.9 IU/L (*P* = 0.0001). Raised GGTP levels were found in 41 (22.2%) individuals with chronic viral hepatitis. Logistic regression analysis demonstrated a significant and independent association between serum GGTP levels and positive HBsAg (*P* = 0.005) and anti-HCV antibody (*P* = 0.0001) status. No significant difference occurred with regard to GGTP values between study and healthy cohorts after correction for age, gender, ethnicity, and viral markers. A subset (*n* = 333) of dialysis patients was tested by molecular technology (branched-chain DNA (bDNA) assay) to measure HCV RNA in serum—an independent and significant association (*P* = 0.0291) between raised GGTP levels and detectable HCV RNA in serum was found [22].

In a large cohort of patients on long-term dialysis in the USA (13,664 patients) after adjustment for several covariates of MCS (malnutrition-inflammatory complex syndrome), an independent relationship between anti-HCV seropositive status and serum alkaline phosphatase activity was found, OR, 1.01 (95% CI, 1.0–1.02), *P* = 0.001 [23]. Among HCV-infected patients, higher serum intact parathormone levels have been detected, 422 ± 423 *versus *338 ± 356 pg/mL (*P* = 0.0001) which persisted even among African-American patients [24]. These observations have been linked to “hepatic osteodystrophy,” a type of bone disease previously described in nondialysis persons with hepatitis [25].

HCV-infected patients on maintenance dialysis have a higher prevalence of hypoalbuminemia when compared to non-HCV-infected counterparts. A survey from the USA reported a significant difference in serum levels of albumin in their cohort of patients on regular dialysis (*n* = 69, 294): 3.68 ± 0.45  *versus *3.76 ± 0.41 g/L (*P* = 0.0001) [23]. They suggested that the impact of HCV infection on nutritional status and inflammation may be the main cause of cardiovascular mortality in this population. Inflammation associated with chronic infections may contribute to the increased CV death risk in dialysis populations. In addition, multiple studies have reported that HCV is associated with liver steatosis, insulin resistance, and hypoadiponectinemia. In addition to conventional cardiovascular risk factors in dialysis patients such as arterial hypertension, hypercholesterolemia, and hyperhomocysteinemia, HCV infection may be an important factor. This opinion is supported by the notion that traditional risk factors only partially explain the mortality excess in dialysis population.

Dialysis patients have multiple comorbidities (arterial hypertension, gastrointestinal bleeding, anaemia, and failure of the vascular access, among others), and clinicians frequently neglect these biochemical alterations in the everyday clinical practice. According to the data from two registries, the Lombard Dialysis and Transplant Registry (RLDT) and the US Renal Data System (USRDS), 4,196 patients on renal replacement therapy were studied—the frequency of cirrhosis ranged between 1.5% and 2.0% [26].

## 4. Impact of Hepatitis C on Survival in Dialysis: Liver-Related and Cardiovascular Mortality

There are extremely limited data available on patient survival in HCV-infected CKD patients who are not on dialysis [27–39]. It has been assumed that survival in the majority of patients with CKD stage 1 and most patients with CKD stage 2 is not significantly different from that of the general population with normal kidney function. For patients with CKD stages 3 and 4, 5-year survival in individuals without HCV infection has been reported to range between 76% and 54%. Increasing evidence on the relationship between anti-HCV positive serologic status and survival in patients on long-term dialysis has been accumulated. A novel meta-analysis including fourteen observational studies (*n* = 145, 608 unique patients on long-term dialysis) demonstrated that anti-HCV positive serological status was an independent and significant risk factor for death in patients on maintenance dialysis [40]. The summary estimate for adjusted relative risk (all-cause mortality) was 1.35 with a 95% confidence interval (CI) of 1.25–1.47. The negative impact of HCV on all-cause survival in dialysis population is in keeping with other sources. The Dialysis Outcomes and Practice Patterns Study (DOPPS), a prospective observational study of representative samples of haemodialysis patients in France, German, Italy, Japan, Spain, the United Kingdom, and the United States (16,720 patients followed up to 5 years) reported an independent and significant association between anti-HCV positive serologic status and mortality (RR, 1.17; *P* < 0.0159) [41].

The same meta-analysis gave information on disease-specific related deaths among dialysis patients according to HCV status [40]. In fact, a stratified analysis showed that the adjusted relative risk (aRR) for liver disease-related death was 3.82 (95% CI, 1.92; 7.61); heterogeneity statistics, *R*_*i*_ = 0.58 (*P* value by *Q* test = 0.087). The adjusted RR for cardiovascular mortality was 1.26 (95% CI, 1.10; 1.45); no heterogeneity was found. In most studies included in this systematic review, the major complications of HCV-related chronic liver disease (cirrhosis and hepatocellular carcinoma) were significantly more frequent causes of death in anti-HCV antibody-positive patients on dialysis than in anti-HCV antibody negative patients on dialysis. These results are consistent with evidence from other sources. According to pooled data provided by the US and RLDT registries, the occurrence of cirrhosis is significantly associated with lower survival in dialysis population (*P* = 0.03) [26].

That the predominant cause of death in patients on regular dialysis is cardiovascular is well known from the 1990s [42], but we observed the persistence of an increased HCV-associated cardiovascular risk after adjustment for several covariates including age, gender, time on dialysis, and diabetes mellitus. Several lines of evidence are in keeping with this observation; Kalantar-Zadeh et al. [23] performed a population-based survey (2,778 patients on long-term dialysis at Da Vita system) and found a hazard ratio for cardiovascular death of 1.43 (95% CI, 1.0–2.06, *P* = 0.05) after adjustment for several case-mix covariates and available surrogates of malnutrition-inflammation syndrome. This was observed over a short (3 years) followup despite the long-term complications of HCV-related liver disease. A significant and favourable impact of kidney transplantation on the adjusted risk of cardiovascular death has been observed in a large cohort of HCV-infected patients with chronic kidney disease by Roth et al. [43]. They retrospectively identified 230 HCV-infected patients during the transplant evaluation and noted an early (<6 months) and sustained decrease in the risk of cardiovascular death (HR, 0.20; 95% CI, 0.08–0.47, *P* < 0.001) after transplant over remaining on the waitlist.

The link between CV mortality and HCV infection among patients receiving long-term dialysis has been studied by Oyake and colleagues [44] using pulse-wave velocimeter measurements. They prospectively evaluated 94 dialysis patients (17 being HCV positive) by measurements of aortic stiffness (by carotid-femoral pulse wave velocity). Multiple logistic regression analysis found that mean blood pressure and HCV viraemia (OR, 9.7; 95% CI, 1.18–81.2; *P* < 0.05) were significantly and independently associated with high pulse wave velocity. Kaplan-Meier survival curves for cerebrovascular and cardiovascular event-free rates indicated a highly significant difference between HCV RNA-positive or negative patients on regular dialysis (log-rank test, *P* < 0.05). The authors suggested an atherogenic role by HCV through aggravation of metabolic syndrome and dyslipidemia [44].

## 5. Impact of HCV on Dialysis: Quality of Life

It has been recently suggested that one of the mechanisms of increased mortality in HCV-positive patients is related to an impairment of quality of life. Numerous investigators have noted that the QoL is lowered in dialysis population; also, HCV-infected individuals with intact kidney function have an impairment of QoL scores. To date, Afsar et al. [45] are the investigators who addressed better this point; they studied 165 patients on regular dialysis (83 anti-HCV positive). They evaluated QoL by SF-36, a test which consists of 36 items, assigned to eight subscales, that can be summarized by a physical component summary score and mental component score. SF-36 has been commonly used and validated in CKD population. There was an independent and significant association between anti-HCV positive serological status and lower mental component summary score (*B*, −3.423, *P* = 0.016). No association between HCV and physical component summary score was found (NS). The presence of symptoms of depression might be one explanation—depression is common in patients with HCV as a reactive phenomenon related to the diagnosis (“labeling” effect) and concerns over long-term health. Depression may be secondary to symptoms such as fatigue and cognitive impairment that can be commonly noted in HCV-infected subjects. In addition, HD treatment is *per se* independently associated with an increased prevalence of depression, which in turn negatively affects health-related QoL.

The quality of life in treated and untreated patients on regular HD with chronic HCV infection was addressed by Akyuz et al. [46]; they treated fifty-five patients with IFN*α*-2b for a median 48 months before administration of the questionnaire (30 received IFN for 6 months; 25 for 12 months), and 40 untreated patients were the control group. All patients were evaluated by the Short Form-36 (SF-36) healthy survey to evaluate their quality of life after antiviral treatment. There was a negative relationship between IFN and health perception (*P* = 0.014; *r* = −0.23). General health perception scores were positively and slightly increased in IFN responder patients, but the difference was not statistically significant compared to nonresponders (*P* > 0.05). Mild and severe physical activity were lower in IFN-treated patients (*P* = 0.0028 and *P* = 0.001, resp.).

It remains unknown whether treatment of HCV in patients on maintenance haemodialysis can decrease mortality by improving QoL, irrespective of biological markers (as an example, liver histology or viral characteristics); thus, dialysis patients with advanced liver disease could still benefit from antiviral therapy in terms of QoL and therefore lowered mortality.

## 6. Antiviral Treatment of HCV in Dialysis: Background and Rationale

Unfortunately, all major randomized controlled trials (RCTs) for the treatment of HCV infection have specifically excluded patients with abnormal kidney function. Accordingly, the available data that critically evaluate the indications for treatment and determine the most efficacious and safe treatment protocols in CKD patients are limited. The KDIGO work group suggested that all CKD patients with HCV infection be evaluated for antiviral treatment. The decision to treat HCV infection in the CKD patients should be based on the potential benefits and risks of therapy, including life expectancy, candidacy for kidney transplantation, and comorbidities. The patients should be appropriately informed of the risks and benefits of antiviral therapy and should also participate in the decision-making process.

The quality of evidence on efficacy and safety of IFN-therapy for hepatitis C in CKD patients is very low. The size of the study group was appropriate in many trials, and some RCTs have been published on this topic. However, there is concern about the applicability of these results to all dialysis patients, as most of the individuals included in these studies were on the waiting list for kidney transplantation and were younger and probably healthier than the general dialysis population. Also, a minority of studies was from North America where many CKD patients are African American. This is of special relevance, because there are racial differences in the response to IFN therapy in patients with intact kidney function.

Life expectancy is a determining factor in advising therapy for HCV-infected patients with CKD. In the context of the lower life expectancy of maintenance haemodialysis patients and the slowly progressive course of chronic HCV infection, long observations periods are needed to observe the benefits of successful antiviral therapy in these patients. Thus, antiviral therapy should not be initiated in CKD patients with a life expectancy lower than 5 years.

The KDIGO Work Group [4] gave recommendation to treat HCV-infected patients accepted for kidney transplantation. The impetus to treat the HCV-infected kidney transplant candidate is different than it is in the general population. Potential benefits of successful antiviral therapy in kidney transplant candidates include slowing the progression of liver disease and reducing the risk of posttransplant complications associated with HCV. The antiviral therapy of HCV-infected kidney transplant candidates is targeted both to treat the disease (slowing the progression of hepatitis C) and the infection (avoiding the extrahepatic complications of HCV after transplant). Positive anti-HCV serologic status after kidney transplantation is implicated in the pathogenesis of acute glomerulopathy [47], *de novo* GN [48–51], new onset diabetes after transplantation [52], excessive exposure to cyclosporine [53], and a higher incidence of chronic allograft nephropathy [54, 55].

Information in support of antiviral therapy of kidney transplant candidates is based on three controlled clinical trials. In the study by Cruzado et al. [56], of 15 HCV-positive recipients who received pretransplant IFN therapy, 10 (67%) had SVR (sustained virological response); only 1 (7%) of these 15 treated patients, who remained viraemic, developed *de novo* GN. Among the 63 untreated HCV-positive allograft recipients, all of whom were RNA viraemic at the time of transplantation, 12 (19%) developed *de novo* GN (*P* < 0.001).

Pretransplant antiviral therapy of HCV-infected transplant recipients appears to lower the incidence of NODAT (new onset posttransplant diabetes mellitus after transplantation). In their controlled trial, Gürsoy et al. [57] observed a higher proportion of NODAT in the group of HCV-positive recipients who had not received IFN than in those who were treated with IFN before transplantation, 25% (10/40) *versus* 7% (1/14), *P* = 0.009.

In a cohort of 50 kidney transplant recipients, a higher frequency of nontreated controls developed chronic allograft nephropathy compared with IFN-treated patients, 41% (13/32) *versus* 6% (1/18), *P* = 0.009. In the logistic regression analysis, the absence of IFN therapy before kidney transplantation was a risk factor for chronic allograft nephropathy with an odds ratio of 12 (*P* = 0.02) [58].

## 7. Antiviral Treatment of HCV in Chronic Kidney Disease: Recommended Schedule and Potential Adverse Reactions

In RCTs of HCV-infected patients with intact kidney function, the highest overall SVRs to date have been achieved with the combination of weekly subcutaneous injections of pegylated IFN and oral ribavirin. This represents the current standard of care for HCV infection, according to the current AASLD (American Association for the Study of Liver Diseases) guidelines. This recommendation is based on the results of three large randomized trials that were completed in IFN-naïve patients with normal kidney function [59–61].

Significant geographical variability exists in the prevalence of the six major HCV genotypes. Although genotype does not predict the outcome of infection, it has been shown to both predict the probability of response to and determine the necessary duration of therapy. Infections with HCV genotypes 1 and 4 are less responsive to IFN-based therapy and require 48 weeks of treatment. In contrast, genotypes 2 and 3 are far more responsive to treatment and require only 24 weeks of therapy to achieve SVR. HCV genotype 5 appears to have a response similar to genotypes 2 and 3 but requires 48 weeks of therapy. Genotype 6 responds better than genotype 1 but not so well as genotypes 2 and 3. These results have been obtained in patients with HCV infection and normal kidney function [4]. A systematic review of studies addressing antiviral therapy based on conventional interferon in patients on maintenance haemodialysis [62–80] reported that an overall summary estimate for SVR was 37% in the whole group and 30% in those patients with HCV genotype 1 [81].

The level of kidney function in the CKD population plays a crucial role on the pharmacokinetics of antiviral drugs targeted at HCV. Kidney filtration and catabolism have a significant contribution to the clearance of IFN and ribavirin; thus, there is the need to make appropriate dosing adjustment and caution. No data exist in the literature to guide therapy for HCV in patients with CKD stages 1 and 2. However, in patients with a GFR >50 mL per min per 1.73 m^2^, impaired kidney function does not have a major impact on the efficacy and safety of combined IFN and ribavirin therapy. As such, the results reported in patients with normal kidney function treated with pegylated IFN plus ribavirin should apply to CKD stages 1 and 2. Limited data exist about combination antiviral therapy (conventional or pegylated IFN plus ribavirin) in CKD stages 3–5 patients. Some information about the use of monotherapy with pegylated interferon in dialysis populations exists [82–87], and the available data on combined therapy (conventional or pegylated IFN plus ribavirin) in the CKD population derive mostly from studies of patients on maintenance haemodialysis [88–96]. There is limited information on the clearance of IFN in patients with CKD stages 3 and 4. Available evidence indicates that there is impaired clearance of standard IFN in patients on maintenance haemodialysis. Therefore, it would be reasonable to assume that IFN clearance might be reduced in patients with advanced CKD not yet on dialysis requiring a dosage adjustment. Reduced kidney function (estimated GFR <60 mL per min per 1.73 m^2^) in CKD stages 3 and 4 would be expected to worsen the side effects of combined antiviral therapy with IFN and ribavirin (Table 1).

The major side effects of *α*-IFN can be flu-like symptoms, malaise, myalgia, asthenia, loss of weight, cardiovascular disorders, haematological abnormalities, or neurological disorders. To date, two different peg-IFNs are available, that is, peg-*α*2a-IFN (Pegasys, Hoffmann-La Roche, Basel, Switzerland) and peg-*α*2b-IFN (Peg-Intron, Schering-Plough, Berlin, Germany). The pegylation of *α*-IFN results in an increased half-life. According to a two-compartment model in patients with normal and reduced kidney function, some authors have studied the pharmacokinetics of ribavirin and found that the probability of response to ribavirin increases with increasing ribavirin concentration. Such approach has been hampered by the limited availability of the assay (high-performance liquid chromatography) to measure steady-state ribavirin levels [97]. Combination antiviral therapy (IFN plus ribavirin) has not been recommended in prior guidelines [98]. According to preliminary findings [88–96], ribavirin should be used in patients on maintenance haemodialysis in a cautious and well-monitored setting. Ribavirin use, indeed, is limited by haemolytic anaemia that can be particularly dangerous in patients with chronic kidney disease, who often have anaemia as well as other comorbidities (e.g., cardiac ischemia) at baseline. The following precautions have been suggested: (1) very low ribavirin dose (200 mg daily or 200 mg thrice weekly); (2) weekly monitoring of haemoglobin levels; (3) high dose of erythropoietin to treat anaemia; (4) low-dose iron to boost erythropoietin therapy. Severe chronic haemolysis can be also responsible for iron overload, liver iron deposition, and acceleration in liver fibrosis progression.

## 8. Antiviral Treatment of HCV in Dialysis: Current Schedules

According to the KDIGO guidelines [4], monotherapy with standard interferon is the therapy of choice for HCV-infected subjects on maintenance dialysis. As demonstrated in a recent meta-analysis, the viral response to monotherapy with standard interferon in maintenance haemodialysis patients is higher than that observed in subjects with chronic hepatitis C and intact kidney function (37% *versus* 7%–16%) [81]. Several mechanisms account for the relatively higher response to IFN in patients undergoing maintenance haemodialysis. Dialysis patients with HCV usually have a lower viral load [21]; the infection is frequently associated with milder forms of histologic liver disease [99]; clearance of IFN is lower in dialysis patients than in non-CKD patients [100]; an increase in endogenous IFN release from circulating white blood cells during haemodialysis sessions has been shown [101]. A marked and prolonged release of hepatocyte growth factor (or other cytokines) caused by haemodialysis could play an additional role [102].

The benefits and risks of antiviral therapy with IFN-based regimens in HCV-infected patients on maintenance haemodialysis have been evaluated in several studies. The quality of evidence in this area is low overall. It has been suggested that tolerance to IFN is lower in dialysis than in non-CKD patients with chronic hepatitis C. Also, the profile of side effects to IFN therapy in dialysis patients seems different from normal controls. In addition to flu-like symptoms, other common side effects leading to interruption of IFN therapy in CKD population are neurologic and cardiovascular disorders. Nevertheless, approximately one-third of haemodialysis patients with chronic hepatitis C have obtained SVR with standard IFN monotherapy [81]. What we need now is to understand whether successful antiviral therapy translates into longer survival in this population.

Tolerance to IFN monotherapy appears lower in patients on maintenance haemodialysis than in non-CKD individuals. The summary estimate of drop-out rate was 17% in dialysis patients who received standard IFN monotherapy, whereas the frequency of side effects requiring IFN discontinuation ranged between 5% and 9% in non-CKD patients with chronic hepatitis C who received a usual dose of standard IFN monotherapy (3 MU thrice weekly for 6 months) [103–105]. The altered pharmacokinetic parameters of IFN in the haemodialysis population, higher age, and high rate of comorbid conditions may, to some extent, explain the higher frequency of side effects leading to IFN discontinuation. The half-life of interferon-*α* was longer in dialysis patients than in normal controls, 9.6 *versus* 5.3 h (*P* = 0.001), and the area under the curve was twice that of patients with normal kidney function [100].

A minority of studies have evaluated combined therapy (pegylated IFN plus ribavirin) in patients on maintenance haemodialysis (Table 2). The quality of evidence on this point is extremely low. The results provided in some studies have been encouraging in terms of efficacy and safety, but the limited size of the study groups does not allow definitive recommendations. Combination antiviral therapy (interferon plus ribavirin) represents clearly an advance, in terms of viral response, compared to monotherapy with standard or pegylated IFN in patients on long-term dialysis; it remains unclear whether combination antiviral therapy based on pegylated IFN use is superior to standard IFN plus ribavirin in dialysis patients with HCV.

The efficacy and safety of combination antiviral therapy (conventional or pegylated interferon plus ribavirin) in dialysis patients with chronic hepatitis C was evaluated by performing a systematic review of the literature with a meta-analysis of clinical studies [106]. The primary outcome was sustained virological response (SVR) (as a measure of efficacy); the secondary outcome was drop-out rate (as a measure of tolerability). 10 clinical studies (151 unique patents) were identified, one (10%) of which was a controlled clinical trial. Most (97.4%) patients were on long-term haemodialysis. The summary estimate for SVR and drop-out rate was 56% (95% CI, 28–84) and 25% (95% CI, 10–40), respectively. The most common side effects requiring interruption of treatment were anaemia (26%) and heart failure (9%). These results occurred irrespective of the type of interferon (conventional or peg-IFN, peg-IFN *α*-2a or *α*-2b), trial design (controlled or cohort studies), or clinical characteristics of patients (naïve, relapsers, or nonresponders). The studies were heterogeneous with regard to SVR and drop-out rate.

## 9. Antiviral Treatment of HCV in Dialysis: Future Perspectives

Novel improvements in the understanding of the viral cycle, and the characterization of viral enzymes which are potential targets, resulted in the development of new molecules, direct-acting antiviral (DAA) drugs targeted against HCV, either specific for genotype 1 (NS3/NS4A protease inhibitors and NS5 polymerase inhibitors) or with wider spectrum (NS5A or entry inhibitors) and nonspecific antivirals (new interferons, cyclophilin inhibitors).

Telaprevir and Boceprevir are two new potent protease inhibitors that have been recently licensed from the Food and Drug Administration (FDA). Both of these drugs inhibit the HCV nonstructural (NS) protein 3-4A serine protease, and recent studies have shown significantly higher SVR rates in patients with genotype 1 infection [107, 108]. For treatment of genotype 1 HCV infection, novel guidelines now recommend their use in combination with pegylated IFN and ribavirin (triple therapy) in treatment-naïve and treatment-experienced patients with genotype 1 chronic HCV infection [109]. Some limitations of triple therapy have been already emphasized including safety (cutaneous rash or anaemia), drug interactions, cost, compliance, and viral resistance [110]. Unfortunately, there are as yet no published studies evaluating the role of triple therapy in the transplant population or in patients with CKD.

## Figures and Tables

**Table 1:** Recommended treatment of HCV infection in patients with chronic kidney disease.

Stage of CKD	IFN	Ribavirin
1 and 2	Pegylated IFN *α*-2a: 180 *μ*g weekly by subcutaneous route Pegylated IFN *α*-2b: 1.5 *μ*g/kg^−1^ weekly by subcutaneous route	800–1200 mg day^−1^ in two divided doses (by oral route)

3 and 4	Pegylated IFN *α*-2a: 135 *μ*g weekly by subcutaneous route Pegylated IFN *α*-2b: 1.0 *μ*g/kg^−1^ weekly by subcutaneous route	Stage 3: 400–800 mg day^−1^ in two divided doses (by oral route) 200–400 mg daily (by oral route) for eGFR <50 mL/min per 1.73 m^2^

5	Pegylated IFN *α*-2a: 135 *μ*g weekly by subcutaneous route Pegylated IFN *α*-2b: 1.0 *μ*g/kg^−1^ weekly by subcutaneous route	200–400 daily (by oral route)

5D	Standard IFN *α*-2a: 3 mU thrice weekly by subcutaneous route Standard IFN *α*-2b: 1 *μ*g kg^−1^ weekly by subcutaneous route or Pegylated IFN *α*-2a: 135 *μ*g weekly by subcutaneous route Pegylated IFN *α*-2b: 1 *μ*g kg^−1^ weekly by subcutaneous route	200 mg daily or 200 mg thrice weekly (by oral route)

**Table 2:** Pegylated IFN plus ribavirin in dialysis patients: baseline characteristics and outcomes of studies.

Authors	SVR	Country	Reference year
Bruchfeld et al.	50% (3/6)	Sweden	2006
Rendina et al.	97.5% (34/35)	Italy	2007
van Leusen et al.	71% (5/7)	Netherlands	2008
Carriero et al.	28% (4/14)	US	2008
Al-Saran et al.	70% (7/10)	Saudi Arabia	2009
Hakim et al.	5% (1/20)	US	2009
Liu et al.	60% (21/35)	Taiwan	2009
Giguere et al.	73% (16/22)	Arab Emirates	2011
Deltenre et al.	50% (16/32)	France	2011

Results have been calculated according to an intention-to-treat (ITT) analysis.

SVR: sustained virological response.

## Abbreviations

AASLD: American Association for the Study of Liver Disease
CKD: Chronic kidney disease
CV: Cardiovascular
GFR: Glomerular filtration rate
GN: Glomerulonephritis
HBV: Hepatitis B virus
HCV: Hepatitis C virus
HIV: Human immunodeficiency virus
DAA: Direct acting antiviral
HD: Haemodialysis
IFN: Interferon
NODAT: New onset diabetes after transplantation
QoL: Quality of life
RCT: Randomized controlled trials
SVR: Sustained virological response.
